# Early-stage knee OA induced by MIA and MMT compared in the murine model via histological and topographical approaches

**DOI:** 10.1038/s41598-020-72350-7

**Published:** 2020-09-22

**Authors:** Gaetan Aüllo-Rasser, Erick Dousset, Sandrine Roffino, Hassan Zahouani, Roger Lecurieux-Clerville, Jean-Noël Argenson, Patrick Chabrand

**Affiliations:** 1 CNRS, ISM UMR 7287, Aix-Marseille University, 13009 Marseille, France; 2 Department of Orthopaedics and Traumatology, APHM, Institute for Locomotion, Sainte-Marguerite Hospital, 13009 Marseille, France; 3 RLC Systems & Research, 13008 Marseille, France; 4 Laboratoire de Tribologie et de Dynamique des Systèmes, 69134 Ecully, France

**Keywords:** Cartilage, Osteoarthritis

## Abstract

Osteoarthritis (OA) is a common degenerative disease whose early management includes promising mechanical treatments. New treatments are initially validated using an animal model in which OA is induced. The MMT (mechanical induction) and MIA (chemical induction) models of OA induction are widespread, but their use to generate early OA is poorly documented. We analyzed and compared early-stage knee OA-induction via these two methods in 16 rats divided into two groups. After 4 weeks of induction, the knees were sampled and studied using both histology (Toluidine Blue and Sirius Red) and surface topology, an innovative technique for characterizing osteoarthritic cartilage. The Mankin-modified score confirms that the two OA-induction models evolved at the same speed. At this early stage, the two models can be differentiated morphologically, although no significant differences were revealed by either cellularity or birefringence analysis. However, the topological analysis generated two forms of quantitative data, the deformation ratio and the cohesion index, that differentiated between the two groups. Thus, the early-stage OA induced by these two models is revealed to differ. The patterns of cartilage damage induced point to MMT as the better choice to assess mechanical approaches to clinical OA treatment.

## Introduction

Knee osteoarthritis (OA) is a common pathology, leading to chronic disability. Its prevalence increases with age: up to 80 percent of people over the age of 65 suffer from OA in high-income countries^1^. There is no treatment that allows patients to fully recover from their loss of cartilage. Orthopaedic surgeons have drawn attention to a gap to be filled in the management of OA^2,3^. Early OA management could open the way to treatments that focus on maintaining or even regenerating cartilage at a still-reversible stage of the pathology. In addition, mechanical treatments for OA, like bone surgery, unloading devices, or distractors, aim to restore a healthy mechanical environment that may stop cartilage degradation^4–6^.

Because the early stages of OA are asymptomatic, obtaining samples of human cartilage is difficult. Furthermore, such samples could not be used to study the potential therapeutic effects of a treatment, since the cartilage would be extracted from its environment. Thus, the animal model remains one of the best ways of assessing the therapeutic benefit of any treatment before human tests. Although some species spontaneously generate OA, induction models allow for better reproducibility and quicker OA development^7^. For the small animal, the well-documented murine model is a good choice for the analysis of OA, recommended by the Osteoarthritis Research Society International^8,9^. Of the different approaches available, the two most common are chemical and mechanical OA induction^9^.

The chemical induction method most frequently used with the murine model involves an intracapsular injection of monoiodoacetate (MIA). MIA is a blocker of chondrocyte aerobic glycolysis that induces apoptosis. The death of chondrocytes makes it impossible to maintain the extra-cellular matrix, which will deteriorate over time. OA induction by MIA is indicated when exploring OA-related pain and is often recommended for pain-related studies^9^. The technique is fast ($\thicksim 5$ min) and dose-dependent. However, the morphological changes induced are reported not to be comparable to OA lesions for human late-stage OA^9^. To the best of our knowledge, analysis has not yet been extended to early-stage OA.

One of the mechanical techniques applied to the murine model is the Medial Meniscal Transection (MMT), used to mimic a post-traumatic OA degradation. The technique consists in cutting the meniscus into two parts, anterior and posterior, resulting in instability in the joint. This leads to atypical stress profiles that will damage the cartilage, an approach that better reflects the conditions for the appearance of secondary OA in humans. However, a surgical procedure is required, including a short recovery period for the animal.

Many studies that jointly assess these two models focus on the pain component of osteoarthritis^10–13^. The two models are also used simultaneously to assess the analgesic^14^ or therapeutic^15,16^ potential of anti-OA drugs. To the best of our knowledge, however, only 3 studies have been devoted to the comparison of the intrinsic effects produced by each method. Jacobs et al.^17^ studied rodent dynamics as a function of the induction method. Pires-Fernandes et al.^18^ used the MIA and MMT models to compare two OA scoring methods. Finally, Thote et al.^19^ compared these two models using a high-performance technique based on micro-scan imaging (EPIC-mCT), at an early stage of OA.

In the clinic, cartilage has the potential to regenerate if it is treated in time. Once OA reaches an advanced stage, the cartilage no longer exists and the only alternative is a replacement prosthesis (UKA, TKA). Furthermore, as MMT is a challenging surgery, we wanted to emphasize that taking the risk of performing surgery rather than an injection is justified by a different pathological behaviour in the early stages, and might lead to false negatives in evaluations of new therapeutic strategies. Thus, the aim of our study was to characterize and compare these two classical osteoarthritis models through a double histological analysis (Toluidine Blue for proteoglycans and Red Sirius for collagen network), as well as to characterize micro-scale surface topology. This yielded both a qualitative and a quantitative picture of early OA effects.

## Results

### Histological analysis

Since the morphological analysis and Mankin score did not distinguish between healthy samples from the MMT group and those from the MIA group, the healthy group consisted of 4 randomly selected samples.

#### Mankin-modified score

Comparison of the Mankin-modified scores, reported in Table 1, of each group showed that the healthy group had a significantly lower degree of OA (Healthy/MMT : $p = 0.03$; Healthy/MIA : $p = 0.01$) than the OA groups. The differences between the MIA and MMT groups were not significant, indicating a comparable degree of OA.

**Table 1:** Mean (and standard deviation) of Toluidine Blue analysis markers—Mankin-modified score and profile line intensity—for each experimental group.

Group	Profile line intensity	Mankin score
Healthy	88.3 (16.74)	1.9 (0.38)
MMT	138.6 (33.41)	3.8 (0.63)
MIA	138.4 (17.93)	5.0 (1.48)

Measurements of intensity were taken on the major defects.

#### Morphology and staining

**Figure 1:**
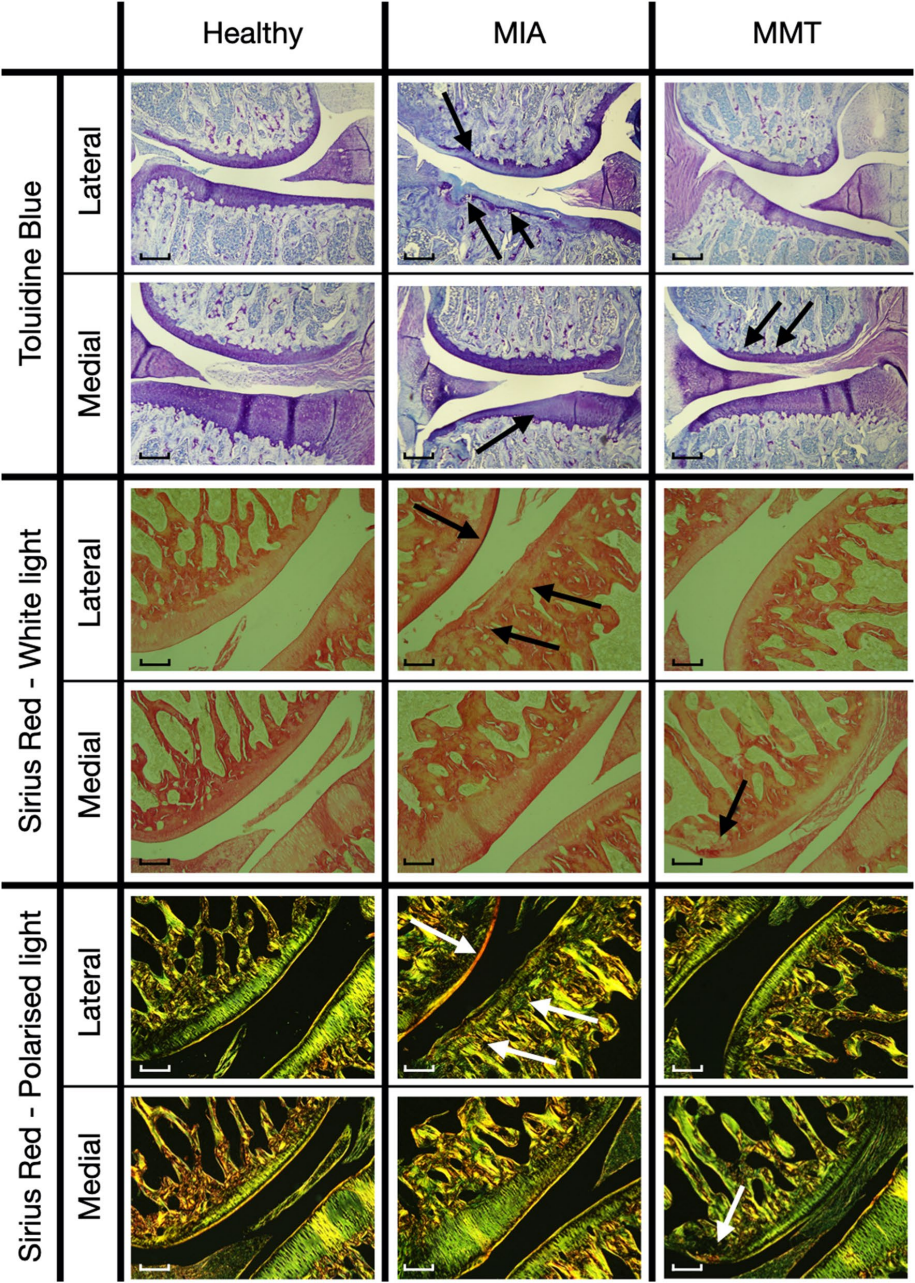
Typical MIA and MMT effect on the cartilage with Toluidine Blue and Red Sirius stainings. On Toluidine Blue slices, MIA shows a tibial cartilage loss of intensity with a loss of proteoglycans for all three zones, and affects surface integrity. MMT shows a loss of proteoglycans in the outer third of the femoral cartilage. Scale bar = 0.5 mm. On Red Sirius slices, in comparison with healthy samples on white light, MIA shows higher intensity in the superficial zone in femoral lateral cartilage and disorganization in tibial lateral cartilage. MMT shows higher intensity on the outer third of the femoral medial cartilage. A quantitative analysis of intensity for polarized views can be found in Fig. 3. Scale bar = 0.25 mm.

Toluidine Blue slices showed two different early OA profiles depending on whether OA was induced by MMT or MIA, as seen on Fig. 1. Healthy samples showed high-intensity purple staining for both tibial and femoral cartilage. The surfaces were regular and smooth, and no abnormal staining was detected.

For the 4 knees studied in this group, the MIA method shows defects mainly located on the tibial cartilage, without medial/lateral preference, in the form of a loss of intensity of toluidine blue staining. 1 knee out of the 4 has more critical defects (Fig. 1, Mankin score = 8). There is damage to the integrity of the cartilage surface, as well as loss of staining in the femoral lateral cartilage.

For both 4 knees, the MMT method induced a defect in the medial femoral cartilage, on its external third. Neither the medial tibial cartilage nor the lateral compartment shows any visible difference from healthy specimens.

Red Sirius staining, observed through white light, confirmed that proteoglycans loss and collagen network disorganization were spatially related (Fig. 1).

#### Cellularity and Red Sirius-Stained Birefringence intensity quantification

**Figure 2:**
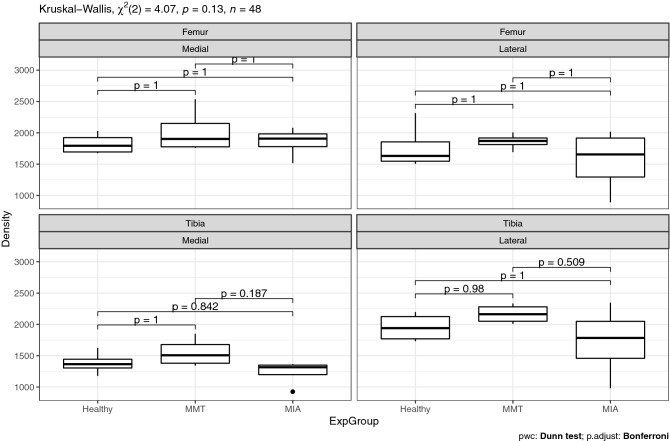
Comparison of the cellularity of each cartilage by experimental group.

**Figure 3:**
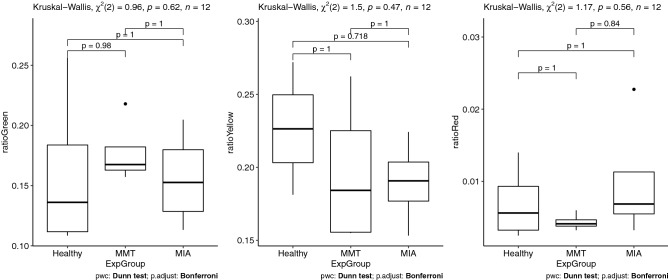
Comparison of the intensity of green, yellow and red fibers under polarized light from Sirius red staining of the collagen network.

Since the morphological analysis showed an influence from induction technique, we chose to analyze results for the whole joint and also by cartilage type, i.e. femoral medial, femoral lateral, tibial medial, tibial lateral. At this early stage of OA, analysis of cellularity (= cell density, Fig. 2) and quantification of birefringence for polarized light red Sirius-stained slices (Fig. 3) did not reveal any statistical differences between groups, neither at the level of the joint nor at the level of a single cartilage.

### Microscale surface topology

In the following analytical techniques, all the healthy samples were compared with each other and with their corresponding pathological samples. Only 3 out of 4 samples from each group could be analyzed, for technical reasons.

#### Deformation ratio

The MIA induction technique generated a deformation ratio along the *y* axis that was statistically different (mean$$ = 0.417$$, $$p = 0.0495$$) from that of the MMT induction technique (mean$$ = 0.205$$). The same trend was found for the *x* axis, without being statistically significant (Table 2).

**Table 2:** Mean (and standard deviation) of the microscale parameters—deformation ratio $\epsilon$ and cohesive index *I* along *x* and *y* axis for each experimental group.

Group	$\epsilon _x$	$\epsilon _y$	$I_x$	$I_y$
$\hbox {MMT}_{Healthy}$	–	–	0.180 (0.079)	0.188 (0.060)
$\hbox {MMT}_{OA}$	0.213(0.250)	0.205(0.048)	0.243 (0.006)	0.397 (0.142)
$\hbox {MIA}_{Healthy}$	–	–	0.207 (0.058)	0.150 (0.092)
$\hbox {MIA}_{OA}$	0.353(0.091)	0.417(0.127)	0.143 (0.081)	0.150 (0.069)

Because of the definition of the deformation ratio, there is no value for healthy samples.

#### Cohesion index

Table 2 shows the mean $I_x$ and $I_y$ for each group. The two healthy groups are comparable. Without being statistically significant, a decrease in $I_x$ is observed for the MIA OA group compared to the MIA Healthy group. The opposite trend is observed for the MMT OA group, with a significative increase ($p=0.0495$) in $I_y$ compared to the MMT Healthy group. A comparison between the two OA groups reveals statistically significant differences for both $I_x$ ($\hbox {p}=0.046$) and $I_y$ ($\hbox {p}=0.046$).

## Discussion

Knee OA is a widespread disease for which there is still no treatment. Current strategies for the management of the disease include mechanical approaches ranging from simple distractors to total arthroplasty. Since early management of the pathology is known to increase the benefits^5^, we sought to determine whether the onset of the pathology could be comparably reproduced using two induction methods, MIA and MMT.

First, the Mankin-modified score allows us to confirm that the rate of OA progression under the two techniques is comparable at 4 weeks post-induction. The cohesion index describes the homogeneity of the tension within the collagen network; it shows that the MIA and MMT control groups can be considered similar. The analysis of cellularity and collagen network by birefringence, an approach that is usually effective for late-OA, does not provide conclusive results, probably because of the early stage of the pathology combined with the limited number of animals.

Morphological evidence under the MIA technique shows a clear impairment of the proteoglycan content of the cartilage. This damage specifically affects the tibial cartilage, with no preference for either medial or lateral compartment, which is in line with Thote’s findings^19^. The deformation ratio shows a strong effect from MIA-induction, with a 41.7% increase in deformation relative to MIA control. A decrease in the relative cohesion index is also observed. This could be explained by the fact that the loss of proteoglycan density decreases the hydration potential of the cartilage, leading to a more relaxed state^20^.

In contrast, the MMT technique leads to localized damage to the femoral cartilage on the outer third of the medial compartment, the site where the healthy meniscus would have acted anyway as a stress distributor. There is no damage to the lateral compartment, which is also coherent with Thote’s^19^. The deformation ratio of the MMT group only increases by 20.5% compared to its control, which is less than half the increase shown by the MIA group.

The loss of proteoglycans is less pronounced than in the MIA group, while the collagen network undergoes greater stress than in the reference state, inducing premature wear. Thus, the MMT group shows greater inhomogeneity of collagen fiber tension, both compared to control and to the MIA samples. This OA-induction technique leads to localized and reproducible cartilage damage and is therefore more appropriate for the analysis of unicompartmental OA.

## Conclusion

The aim of this study was to provide insights into the first stages of OA induction via two of the most common techniques, MIA and MMT, to inform choice when establishing a clinical protocol. Our analysis used both a common histological approach and an innovative approach based on confocal microscopy. At this stage, histology yielded qualitative distinctions between the two induction techniques but could not provide quantitative distinctions of statistical significance. We therefore developed a novel analysis method by studying the surface topology of cartilage at the microscale. This provided information on mechanical properties (deformation ratio, cohesion index) of the collagen network, revealing two opposite patterns of OA degradation. A statistically significant quantification of the mechanical state of the collagen network in early-stage OA was obtained.

Several differences were found between the two models of OA induced. MIA-induced OA is initiated by a diffuse loss of proteoglycan density over the medial and lateral tibial cartilages. The MMT technique, contrastingly, initiates OA via disorganization of the collagen network, and in particular localized wear on the external zone of the medial femoral cartilage, extending over a length virtually identical to the length of the meniscus’s penetration into the joint, i.e. where it would have played its role without induction. This study highlights the importance of the choice of induction technique, even in modeling early OA. At first glance, MIA OA induction might appear to be the obvious choice because of its speed and safety, with no surgery involved and dose-dependance. However, this study shows that from the very first stages of the pathology, symptoms differ from those induced by MMT: there is damage to all the articulations rather than localized damage (to the medial compartment, as in humans), and it occurs through the proteoglycans rather than the collagen network. Also, MIA is known to lead to major loss of cellularity at medium term, making it impossible for treatment to stimulate cartilage regeneration. These findings led us to select MMT for the continuation of our work on developing a medial discharge device for the management of medial gonarthrosis in humans.

## Methods

### Animal and experimental design

16 male Sprague-Dawley rats weighing 350 gr were randomly divided into 2 groups. After two weeks of acclimatization to laboratory conditions, the first group was injected with MIA to induce OA in the left hind leg, while the second underwent a medial meniscal transection (MMT) in the same leg^9^. Weight variation was observed visually throughout the experiment, since none of our methods of analysis required precise monitoring of the weight of the animals, and no variation was observed.

### OA induction methods

The MIA group were orally anaesthetized with isoflurane at 4%, then at 2.5% throughout the intervention. An intra-articular injection of 1 mg of monoiodoacetate in 50 mL of saline solution was performed in the left knee. After even distribution of the solution was ensured by repeatedly bending the limb, the rat was placed in a cage for the recovery phase.

The MMT group rats were anaesthetized via the same procedure as the first group. An incision was made in the left knee, the joint capsule was opened, and the medial meniscus was cut into two anterior and posterior parts, without damage to surrounding cartilage. Finally, the cut was sutured and the rat was placed in a cage for the recovery phase.

After 4 weeks, the rats were euthanized by overdose of sodium pentobarbital (1 mL for 0.5 kg i.p) and both hind legs (osteoarthritic + contralateral) were harvested. 4 analysis groups were composed of rats from MIA group and MMT group. Left knees that underwent OA induction generated two “subgroups”, MIA-OA and MMT-OA, while right knees was used as healthy control, generating two other subgroups, MIA-Healthy and MMT-Healthy. In fact, a study by Jacobs et al.^17^ shows that there is an influence on the gait of rats from the early post-induction period. However, this study uses more aggressive models of osteoarthritis than those we used. The absence of cartilage damage on the contralateral knees observed by histology supports our decision to use these knees as a control group, allowing us to reduce the number of animals used in the experiment. As the topology technique we present is innovative, we do not assume the hypothesis that the two healthy subgroups can be treated as an undifferentiated one. In contrast, this hypothesis is assumed when the paper refers to more classical analysis like histology. Within each group, half of the legs were analyzed by histology, while the second half were analyzed by microscale surface topology^21,22^.

### Histological analysis

**Figure 4:**
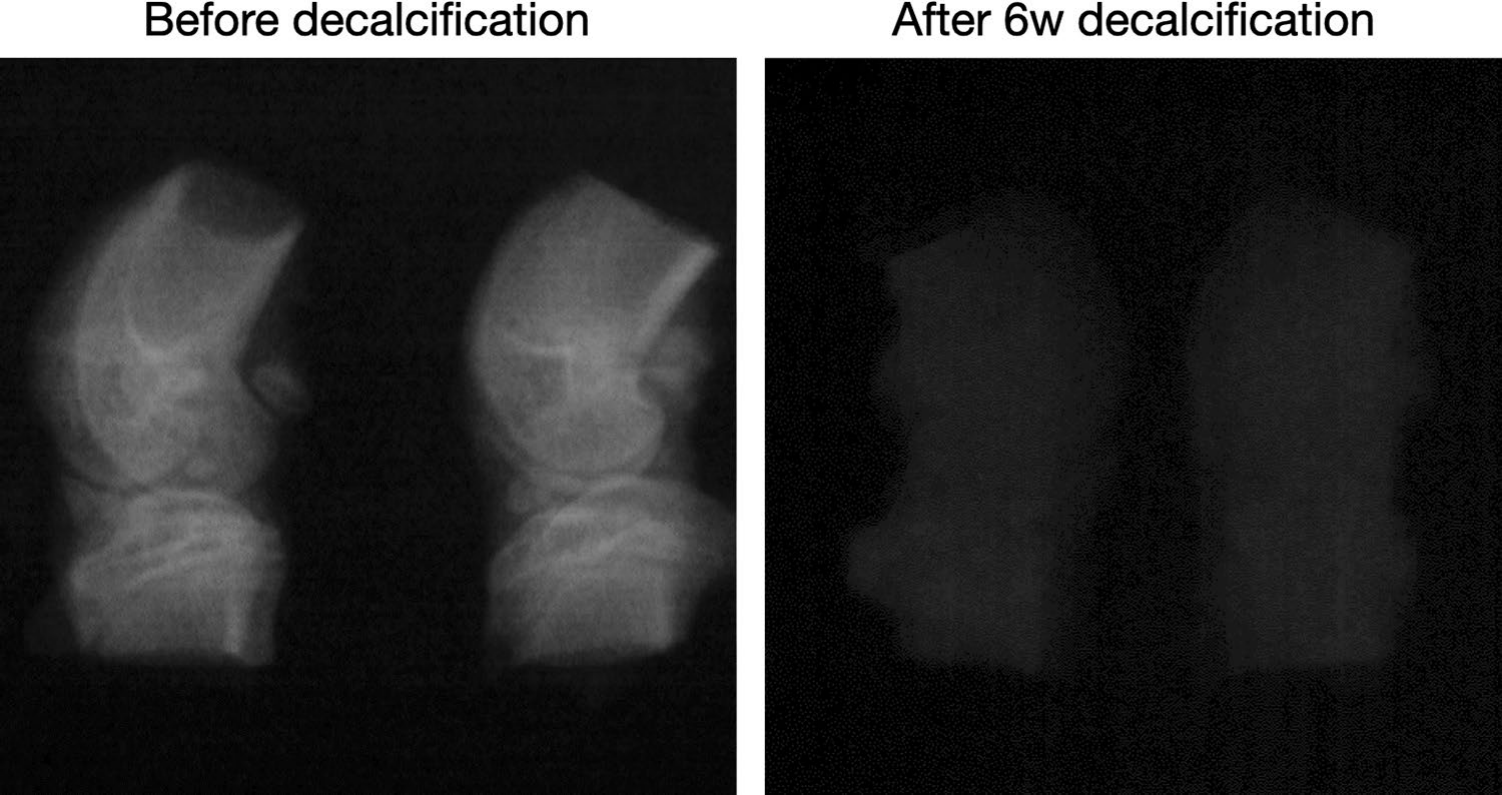
Sample decalcification during histological protocol monitored by X-ray imaging.

After dissection, the knees were cleaned of surrounding tissues and fixed in 4% paraformaldehyde (Merk Millipore, Fontenay sous Bois, France) in 0.01 mol/l phosphate buffer saline (Sigma-Aldrich) at pH 7.4 for 1 week. Samples were rinsed in PBS (pH 7.4) and decalcified in 12% of ethylenediaminetetraacetic acid (EDTA, Sigma-Aldrich) during 6 weeks (Fig. 4). Radiographies were performed to control decalcification. When decalcification was achieved, samples were washed in deionized water, dehydrated in alcohol baths of increasing concentration ($60^{\circ }$, $80^{\circ }$, $95^{\circ }$, $100^{\circ }$), and finally cleaned with tissue clearing agent (Histo-Clear-II, National diagnostics, Atlanta, USA). Knee joints were infiltrated in three successive baths of liquid paraffin during 4 h and embedded in paraffin (polyisobutylene mixture, Paraplast plus, Sigma-Aldrich)^23^.

The blocks were cut according to Gerwin^9^, in the frontal plane into $5 \,\upmu \hbox {m}$ thick sections every $200\, \upmu \hbox {m}$ in the region where menisci are triangle-shaped. Toluidine Blue ($\hbox {pH} = 4.2$) and PicroSirius Red dyes were used to reveal the characteristic structures of the joint cartilage, respectively proteoglycans and collagen fibers. The sections showing the worst damage to each knee were kept for analysis.

**Figure 5:**
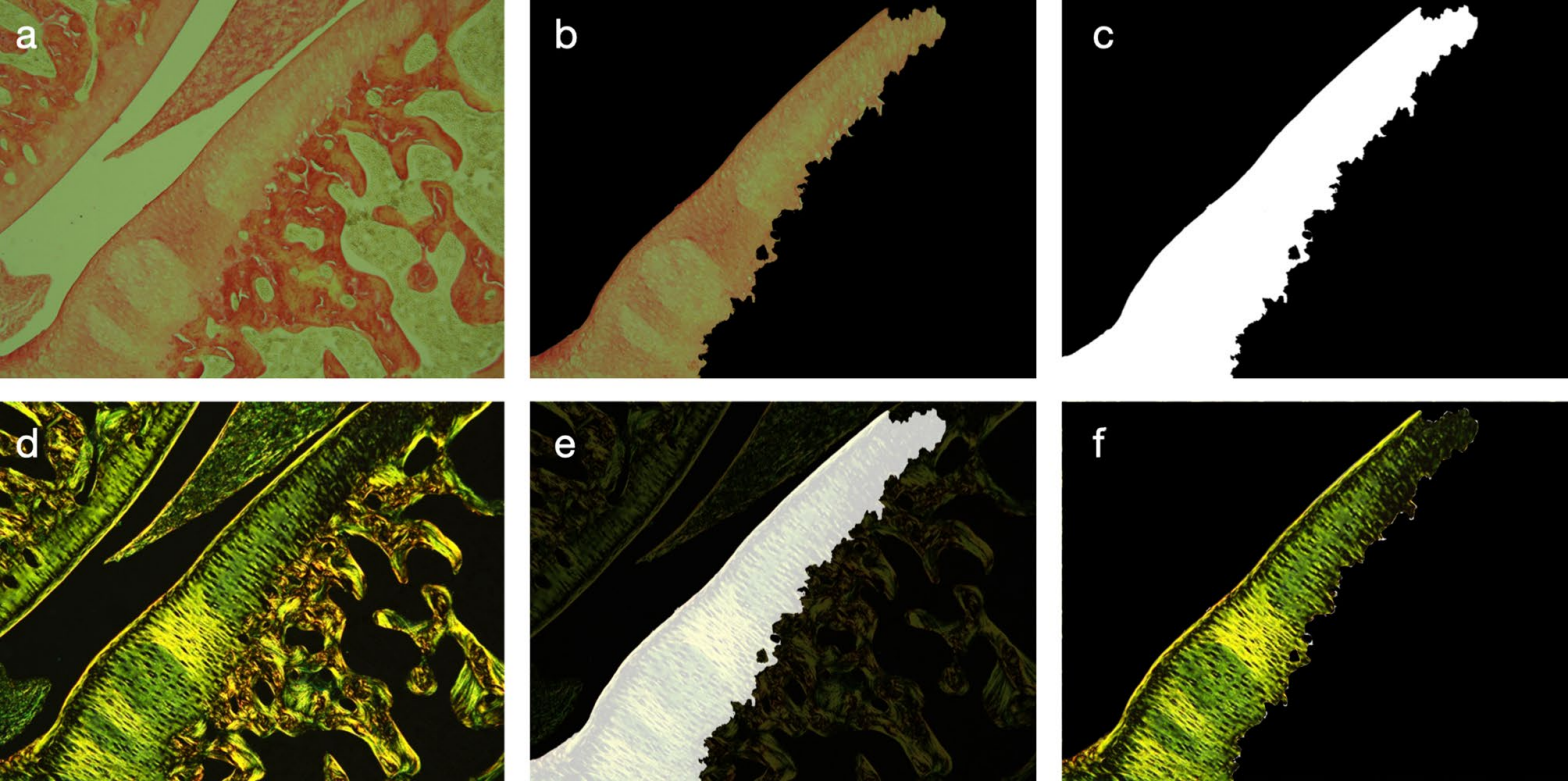
Segmentation of the polarized-light red sirius slices. Segmentation is obtained from white-light pictures, which offer better results and easier handling than polarized light pictures. The slice is placed under the lens of the microscope. Picture is taken with (**a**) and without polarized filter (**d**). Cartilage semi-automatic segmentation is performed on white-light picture (**b**). A mask is made from this segmentation (**b**) and is applied with AND operator (**e**). Segmented polarized cartilage is obtained (**f**) and is used for quantification. All these operations were done on the ImageJ software (ImageJ 1.53c, Wayne Rasband, NIH USA, http://imagej.nih.gov/ij). Scale bar = 0.25 mm.

Slices were observed through an Olympus BX40 optical microscope (x4 lens for morphology and staining, x20 lens for cellularity) and photographed with an Olympus DP21 camera. The Toluidine blue slices were compared and their Mankin-modified score and cellularity computed. The Mankin score is a combined score assessing structure, cells, tidemark integrity and matrix staining. It ranges from 0 points for an healthy cartilage to 14 points for the most severe cartilage lesions^24^. For Mankin-modified scoring and cell counting, each slice was manually analyzed by three different operators and the mean computed. Cell counting was performed by cartilage area to obtain cellularity values. The standard deviation for each case is reported. Cellularity is defined as cellular density. For each cartilage of the joint, pictures were taken with the microscope at a magnification of 200. Three consecutive pictures were needed to cover one whole cartilage (inner, middle and outer zones), and overlap was avoided as far as possible. For each picture, cells were manually counted by three different reasonably experienced operators. The average number of cells was divided by the cartilage area of the picture. The cellularity of a cartilage is given by the average of the three values obtained. For the Picro-Red Sirius slices, collagen fiber birefringence was observed via a linearly polarizing filter (Olympus U-POT polarizer) set to obtain a black background. The polarized-slice pictures were taken at the healthy cartilage maximum birefringence intensity angle, and this angle was reproduced on subsequent slices^25,26^. The ROI, i.e. articular cartilage, was segmented from white light images, using ImageJ software (ImageJ 1.53c, Wayne Rasband, NIH USA, http://imagej.nih.gov/ij). This mask was applied to the polarized light images to isolate cartilage (Fig. 5). Different color thresholds (Red, Yellow, Green) were applied and the selected areas were compared to the total cartilage area. Since the birefringed color reflects the local state of the collagenous network (fiber size, density, spatial arrangement, etc), a variation in this color indicates a change in the network^27^.

### Microscale surface topology analysis

The analysis methods described below are innovative and are based on characterizing the micro-scale surface topology of the articular cartilage. Local variations in the height of the articular cartilage are measured as a wave function, as in standard surface topology analysis, yielding wave formalism that can be used for the characterization^28^. We provide extensive details because, to the best of our knowledge, this is the first time that such a method has been used to analyze osteoarthritic articular cartilage.

#### Sampling

**Figure 6:**
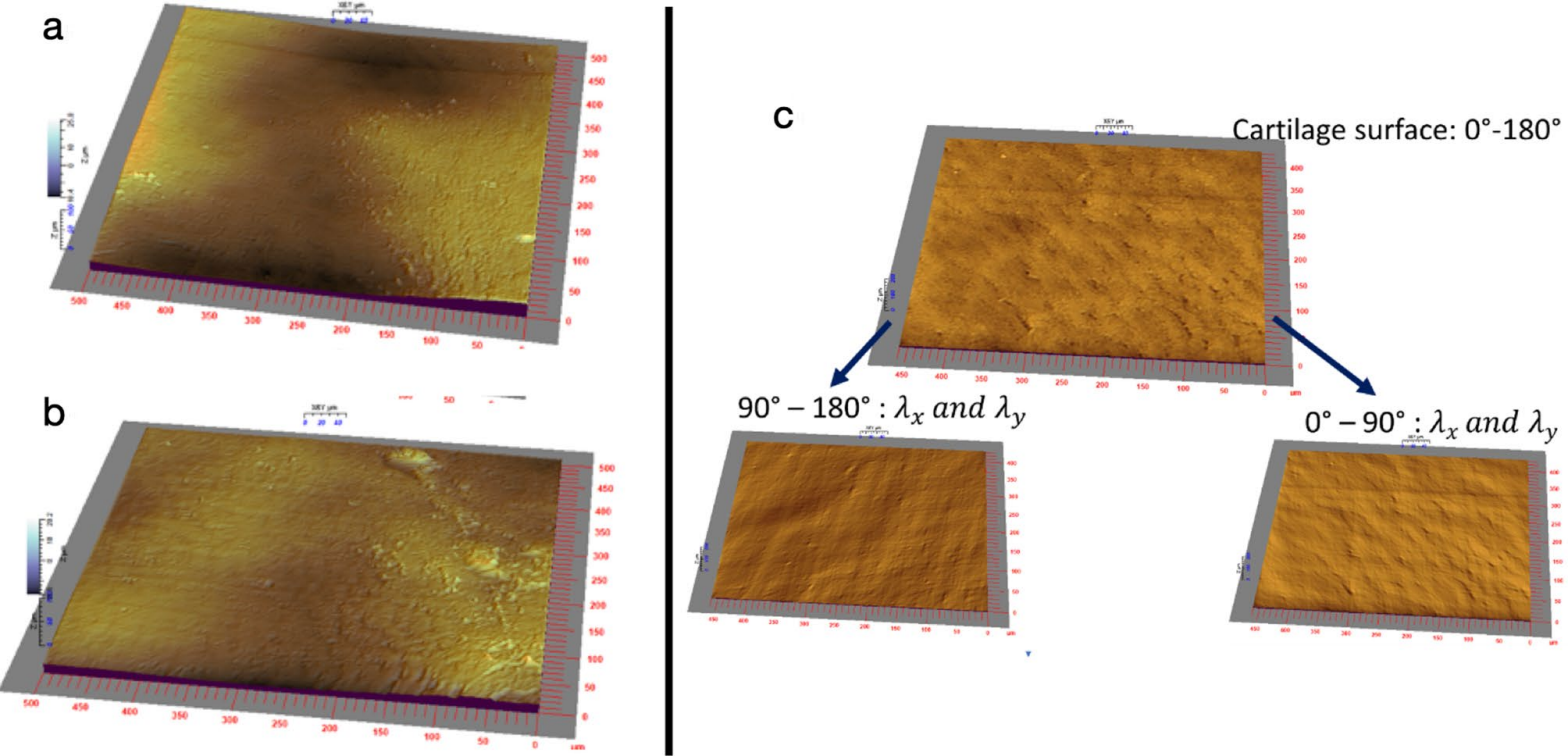
(**a**,**b**) Two examples of OA cartilage reconstruction (sample size : $$512\, \upmu \hbox {m}$$$$*\, 512\, \upmu \hbox {m}$$) directly generated from confocal microscopy, respectively from MMT and MIA samples. Average wavelengths $$\lambda _x$$ and $$\lambda _y$$ for the whole study vary between 8 and $$28 \,\upmu \hbox {m}$$. (**c**) Decomposition of the spatial frequencies of the gradients of the cartilage morphologies.

The surface morphology of the joint cartilage surface was analyzed via a confocal microscope with white light. The confocal microscope exploits the principle of chromatic aberration, with a vertical resolution of 5 nm and a lateral resolution of $1 \,\upmu \hbox {m}$. The image size of each cartilage is 512x512 points and the sampling step in the *x* and *y* directions is $1 \,\upmu \hbox {m}$. For each cartilage surface measured, a three-dimensional reconstruction of the surface is carried out. Figure 6 shows two examples of reconstructed OA cartilage surfaces.

To quantify the effect of each technique on the state of overall mechanical deformation and the state of cohesion of the morphological structure of the cartilage, two quantitative approaches were developed.

- Statistical approach to quantifying MIA effect and MMT effect on overall cartilage deformation: to determine the deformation ratio for healthy cartilage and for cartilage with OA induced by MMT or MIA, statistical analysis was performed on the wavelengths of the maxima of the gradient of cartilage morphology in *x* and *y* directions (*x* was designated as the medio-lateral axis, and *y* as the antero-posterior axis). In general, the lengths between the maxima of the peaks are considered to be very sensitive to the state of aging and damage of living tissue, and provide an indication of the state of deformation induced by MMT or MIA relative to the healthy state considered as the reference state.
  For each image of the cartilage, the gradient of the topography is determined. The wavelengths in the *x* and *y* directions are determined as the average values of distances $L_x$ and $L_y$ between the maxima of the gradient operator.
  The gradient operator of surface heights *z*(*x*, *y*) of the cartilage morphology is defined as:
  1 $$\begin{aligned} \overrightarrow{\nabla z(x,y)} = \begin{pmatrix} \dfrac{\partial z(x,y)}{\partial x} \\ \dfrac{\partial z(x,y)}{\partial y} \\ 1 \\ \end{pmatrix}. \end{aligned}$$
  If we define the average wavelengths *x* and *y* separating the maxima of the gradient in the directions *x* and *y* by:
  2 $$\begin{aligned} \lambda _x = \frac{1}{512}\sum _1^N L_x ; \,\lambda _y = \frac{1}{512}\sum _1^N L_y . \end{aligned}$$
  The average deformation ratios in x and y directions between the OA cartilage and the healthy cartilage are defined as:
  3 $$\begin{aligned} \varepsilon _x(\%) = \frac{ {\overline{\lambda }}_x^{\text { OA}} - {\overline{\lambda }}_x^{\text { Healthy}} }{{\overline{\lambda }}_x^{\text { Healthy}}} ;\, \varepsilon _y(\%) = \frac{ {\overline{\lambda }}_y^{\text { OA}} - {\overline{\lambda }}_y^{\text { Healthy}} }{{\overline{\lambda }}_y^{\text { Healthy}}} \end{aligned}$$
  The deformation ratio is quantified using the wavelengths of healthy cartilage as reference lengths relative to the wavelengths of OA. Statistical wavelength analysis of the surface morphology takes into account the change in the spatial aspect of the topography as a signature of the change in the microstructure after cartilage damage. This yields an OA/Healthy deformation ratio, where a negative deformation ratio indicates cartilage compression, and a positive deformation ratio indicates extension.
- Statistical analysis of the cohesion indices of healthy cartilage and OA cartilage: to determine the cohesion indices of healthy cartilage and OA cartilage, the image of each cartilage was explored by spectral analysis of the morphology of the surfaces. The idea behind this approach is that the state of cohesion of the cartilage is due to the biaxial state of the cohesion forces exerted in *x* and *y* directions on the analyzed area, and whose size is defined by the image analyzed. This state of cohesion will be quantified through the state of non-equilibrium of the wavelengths of the gradient maximum in the Fourier domain along the *x* and *y* axes. The extraction of wavelengths of between $0^{\circ }$ and $90^{\circ }$ ($\lambda _x$ and $\lambda _y$ here) and $90^{\circ }$ and $180^{\circ }$ ($\lambda _x$ and $\lambda _y$ here) from each image is the signature of the effect of biaxial cartilage tension. The Fourier transform in polar coordinates is used to extract two images whose spatial frequencies are between $0^{\circ }$–$90^{\circ }$ and $90^{\circ }$–$180^{\circ }$ defined by
  - In direction *x*:
    4 $$\begin{aligned} \lambda _x^{0 \Rightarrow 90^{\circ }} = \frac{1}{N} \sum _{i=1}^N \lambda _{i,x} \left[ \text {TF}^{-1} (F(\nu , \theta ) \right] _{0 \Rightarrow 90^{\circ }} ; \,\lambda _x^{90 \Rightarrow 180^{\circ }} = \frac{1}{N} \sum _{i=1}^N \lambda _{i,x} \left[ \text {TF}^{-1} (F(\nu , \theta ) \right] _{90 \Rightarrow 180^{\circ }} \end{aligned}$$
  - In direction *y*:
  5 $$\begin{aligned} \lambda _x^{0 \Rightarrow 90^{\circ }} = \frac{1}{N} \sum _{i=1}^N \lambda _{i,x} \left[ \text {TF}^{-1} (F(\nu , \theta ) \right] _{0 \Rightarrow 90^{\circ }} ; \,\lambda _x^{90 \Rightarrow 180^{\circ }} = \frac{1}{N} \sum _{i=1}^N \lambda _{i,x} \left[ \text {TF}^{-1} (F(\nu , \theta ) \right] _{90 \Rightarrow 180^{\circ }} \end{aligned}$$
  with $\text {TF}^{-1}$ the inverse Fourier transform and $\lambda _{i,x}$ and $\lambda _{i,y}$ the wavelengths of the gradients of the cartilage morphologies as defined above, Fig. 6.
  The cohesion indices, which provide an insight into the isotropy of the collagen network, are defined as:
  6 $$\begin{aligned} I_x(\%) = \left| \frac{ \overline{\lambda _x^{0 \Rightarrow 90^{\circ }}} - \overline{\lambda _x^{90 \Rightarrow 180^{\circ }}}}{\lambda _{x_{min}}} \right| ; \,I_y(\%) = \left| \frac{ \overline{\lambda _y^{0 \Rightarrow 90^{\circ }}} - \overline{\lambda _y^{90 \Rightarrow 180^{\circ }}}}{\lambda _{y_{min}}} \right| . \end{aligned}$$
  Cohesion indices quantify the integrity of the cohesion of the same surface: the more cohesive the surface, the closer the cohesion indices are to zero. Cohesion indices have high values when a surface has lost its integrity due to the ageing and/or pathology of the microstructure or after an external action is performed. Thus, if the cohesion forces are balanced in *x* and *y* directions, the cohesion indices will be low values. Otherwise, an anisotropic state of internal cohesion results in a high cohesion index.

### Statistical anaysis

Due to the low number of samples, all the datas were evaluated through a Kruskal–Wallis Test. Dunn tests with Bonferoni correction were used for pairwise comparison. Significance thresholds were set to 95%.

### Ethical approval

Anesthesia and surgical procedures were performed according to French law on animal care guidelines. The Animal Care Committees of Aix-Marseille Université (AMU) and Centre National de la Recherche Scientifique (CNRS) approved the protocols (APAFIS#20453-2019091611408717v2). Individuals conducting the research were listed in the authorized personnel section of the animal research protocol or added to a previously approved protocol (Licence B 13 01306). All experiments complied with the recommendations provided in the Guide for Care and Use of Laboratory Animals (U.S. Department of Health and Human Services, National Institutes of Health) and with the European Community’s council directive of 24 November 1986 (86/609/ EEC), the ARRIVE Guidelines and the U.K. Animal (Scientific Procedure) Act, 1986. All these guidelines were carefully followed. No clinical sign of pain or discomfort (i.e. screech, prostration, hyperactivity, anorexia) or paw-eating behavior was observed throughout the study.
